# Individual Globular Domains and Domain Unfolding Visualized in Overstretched Titin Molecules with Atomic Force Microscopy

**DOI:** 10.1371/journal.pone.0085847

**Published:** 2014-01-20

**Authors:** Zsolt Mártonfalvi, Miklós Kellermayer

**Affiliations:** 1 Department of Biophysics and Radiation Biology, Semmelweis University, Budapest, Hungary; 2 MTA-SE Molecular Biophysics Research Group, Semmelweis University, Budapest, Hungary; University of Leeds, United Kingdom

## Abstract

Titin is a giant elastomeric protein responsible for the generation of passive muscle force. Mechanical force unfolds titin’s globular domains, but the exact structure of the overstretched titin molecule is not known. Here we analyzed, by using high-resolution atomic force microscopy, the structure of titin molecules overstretched with receding meniscus. The axial contour of the molecules was interrupted by topographical gaps with a mean width of 27.7 nm that corresponds well to the length of an unfolded globular (immunoglobulin and fibronectin) domain. The wide gap-width distribution suggests, however, that additional mechanisms such as partial domain unfolding and the unfolding of neighboring domain multimers may also be present. In the folded regions we resolved globules with an average spacing of 5.9 nm, which is consistent with a titin chain composed globular domains with extended interdomain linker regions. Topographical analysis allowed us to allocate the most distal unfolded titin region to the kinase domain, suggesting that this domain systematically unfolds when the molecule is exposed to overstretching forces. The observations support the prediction that upon the action of stretching forces the N-terminal ß-sheet of the titin kinase unfolds, thus exposing the enzyme’s ATP-binding site and hence contributing to the molecule’s mechanosensory function.

## Introduction

Titin (also known as connectin) forms a filamentous scaffold within the muscle sarcomere [1]–[4]. It spans the distance between the middle and the edge of the sarcomere and is tightly bound in the Z-disk, the M-line and the thick filament. Titin is a linear chain of globular (immunoglobulin, Ig and fibronectin, FN) domains interrupted with unique sequences, most notably the unstructured proline (P), glutamate (E), valine (V) and lysine (K)-rich PEVK domain [5]. One of the main functions of titin is the generation of passive tension [6]–[8]. The response of titin to mechanical forces has been quite extensively studied in single-molecule experiments [9]–[11], suggesting that titin behaves as an entropic polymer chain in which mechanical force induces domain unfolding. At low forces the tandem-Ig regions straighten [8], [12]–[14], then at increasing forces the PEVK domain [13]–[16] and, in cardiac titin, the N2B unique sequence [17], [18] are recruited into the elongation process. Finally, the globular domains unfold with a probability that depends exponentially on the applied force and linearly on the time of exposure to this force [9]–[11]. While the global, average structure of titin under force is well described by entropic polymer models [19], little is known about the local, specific structural features. Out of the more than 300 domains comprising titin merely a handful have been characterized for molecular structure [20], and, to our knowledge, only molecular-dynamics simulation data are available that address the high-resolution detail of force-driven structural changes [21]. The mechanical stabilities of a few recombinant globular titin domains have been characterized and compared [22], and it is generally thought that force imposes a temporal order on the domain-unfolding sequence so that mechanically weak domains unfold first [11]. However, whether there is any spatial order in globular-domain unfolding within the context of full-length titin is not known. Thus, the exact structure of titin and the sequence of structural changes under force are outstanding unresolved problems. Ideally, one would like to visualize titin, with as high a resolution as possible, during its extension. Previously, molecular combing was used to visualize, by rotary shadowing and electron microscopy, extended titin molecules [23], [24]. The unfolding of the PEVK domain has been shown in molecules stretched with a putative force of ∼800 pN [24], and even the unfolding of globular domains was inferred [25]; however, further structural insight was limited by the resolution (∼4 nm) of the shadowing method. In the present work we combined molecular combing, driven by a receding meniscus, with high-resolution atomic force microscopy (AFM) imaging, which enabled us to resolve detail, including the presence of individual unfolded and globular domains in overstretched single titin molecules. Based on topographical distance mapping we infer that the unfolded titin region nearest its M-line end is likely part of the kinase domain, which is consistent with prior experimental evidence [26] suggesting that the titin kinase may sense forces via mechanically-driven partial unfolding.

## Materials and Methods

### Preparation of Titin

Skeletal-muscle titin was prepared from rabbit *m. longissimus dorsi* by using previously published protocols [9], [27]. Muscle samples were obtained from male New Zealand white rabbits by using a CO_2_-induced euthanasia procedure (Protocol title: “In vivo imaging methods”; Approval number: XIV-I-001/29-7/2012) approved by the Semmelweis University Regional and Institutional Committee of Science and Research Ethics (Address: Üllői út 93, Budapest 1091 Hungary) and by the Directorate for Food-chain Safety and Animal Health of the Government of Pest County (Address: Lehel u. 43–47., Budapest 1135 Hungary) with reference to the Hungarian Law on the Protection and Humane Treatment of Animals (XXVIII/1998). Purified titin samples were stored on ice in the presence of protease inhibitors (40 µg/ml leupeptin, 20 µM E64) until further use. Typically, samples were used within two weeks of purification. Except where noted otherwise, all chemicals were obtained from Sigma-Aldrich.

### Stretching Titin with Receding Meniscus

**Figure 1:**
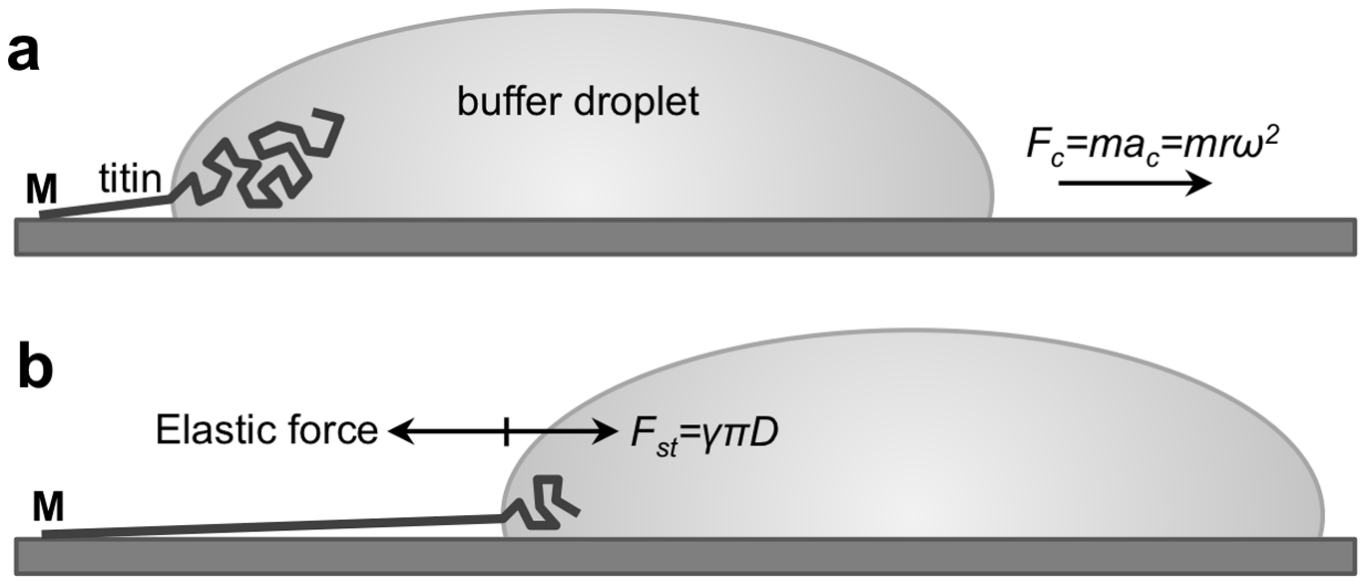
Schematics of the titin-stretch experiment. **a.** A titin molecule, attached by one of its ends (typically its M-line end, indicated with *M*) to the mica surface is pulled by a receding buffer droplet accelerated by centrifugal force (*F_c_*). *m* is droplet mass and *r* is the distance from the center of rotation. **b.** At each time point during droplet movement, a surface-tension(*γ*)-based force (*F_st_*, counteracted by the elastic force borne in the protein chain), proportional to chain diameter (*D*), stretches titin before it is stabilized by binding to the surface.

Titin was extended by molecular combing with receding meniscus based on steps reported earlier [24] (**Fig. 1**). Titin was diluted with PBS solution (10 mM K-phosphate pH 7.4, 140 mM NaCl, 0.02% NaN_3_) containing 50% glycerol to an approximate final potein concentration of 20 µg/ml. In typical experiments urea was added to a final concentration of 1 M to reduce protein aggregation. At this concentration urea was shown not to induce globular-domain unfolding [28]. 20 µl sample was applied to freshly cleaved mica and immediately spun in a custom-built rotor with 13,000 RPM for 10 s. The rotor, a flat round anodized aluminum block, held the mica sheet at a radius of 5 cm from the rotation axis of a tabletop centrifuge. Following spinning, but before the complete drying of the residual liquid layer, the mica surface was extensively washed with distilled H_2_O and dried with clean N_2_ gas. Often the specimen was dried further under ambient conditions prior to AFM imaging. In some experiments the sample was covered with PBS solution immediately after the centrifugation step so as to reveal molecular structure unaffected by dehydration.

### Atomic Force Microscopy and Image Analysis

Titin samples were imaged with a high-resolution atomic force microscope (Cypher, AsylumResearch, Santa Barbara, CA). Dehydrated samples were imaged in tapping mode with a stiff cantilever (Olympus AC160 or AC55TS with nominal tip radii of 9 and 7 nm, respectively) at a line-scan rate of 3–6 Hz and pixel resolution of 0.5 - 2 nm. Hydrated samples were scanned in tapping mode with a soft, high-resonance-frequency cantilever (Olympus BL-RC150VB, BioLever B, nominal tip radius 25 nm) a a line-scan rate of 2–3 Hz and a pixel resolution of 2–4 nm. Images were corrected for flatness of field and color contrast (offset and range) by using built-in algorithms of the AFM driver software. Topographical distance (e.g., filament width) measurements were corrected for tip convolution as published earlier [29]. Accordingly, titin molecule width (*W*) was calculated as
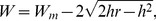
(1)where *W_m_* is measured width at half of the maximum, *r* is tip radius and *h* is topographical height in the filament axis. The width of topographical gaps (*G*) was obtained as
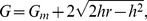
(2)where *G_m_* is the measured gap width.

### Calculations and Statistics

The average stretching force (*F*) on the individual titin molecules was calculated by three different methods. First, by using *a priori* calculation based on surface tension (*γ*) and cross-sectional dimension of titin domains [24] according to
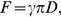
(3)where *D* is diameter of a titin domain in a molecule pulled taught (2 nm, [30]). Second, by using an *a posteriori* calculation based on the ratio of the end-do-end length (*z*) and contour length (*L_C_*) of titin’s globular domain according to the wormlike-chain equation [31] as
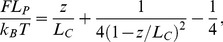
(4)where *L_P_* is persistence length of unfolded protein (0.4 nm [11]), *K_B_* is Boltzmann’s constant and *T* is absolute temperature. For this calculation the unfolded-domain end-to-end length was obtained experimentally, and the average contour length of a titin globular domain was calculated to be 32.1 nm based on the mean amino-acid content of 91.8/domain obtained from protein’s annotated sequence data (http://www.uniprot.org/uniprot/Q8WZ42) and assuming 3.5 Å residue spacing. Finally, by using an *a posteriori* calculation based on the average number of domains unfolded per titin (*N_U_*, obtained experimentally) compared with domain-unfolding probability under mechanical load [32], [33] calcuated as
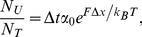
(5)where *N_T_* is the total number of titin’s globular domains (300 [5], [34], [35]), Δ*t* is the time period during which the meniscus travels across the length of a single titin molecule, *α*_0_ is the rate of spontaneous domain unfolding (3×10−5 s−1 [33]) and Δ*x* is the width of the unfolding potential (3 Å [33]). Δ*t* was estimated to be 5×10−6 s based on the centrifugal speed (RPM = 13,000 min−1), rotor radius (r) and average overextended titin length (s) as



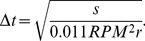
(6)Data were processed with IgorPro (v.6.2.2.2) and KaleidaGraph (v.4.2) program packages.

## Results

**Figure 2:**
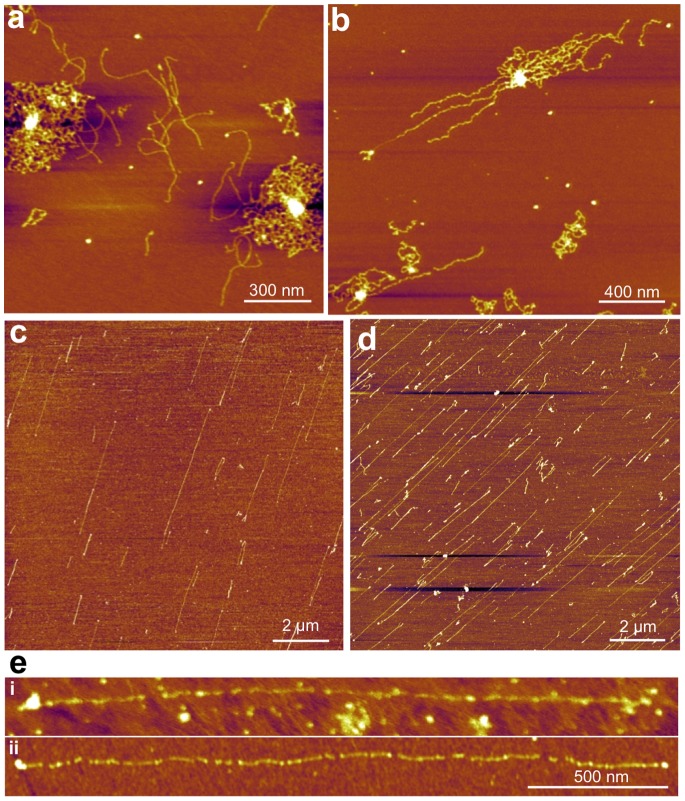
Titin molecules at different locations on the mica surface. **a.** Place of sample application near the center of the mica surface. **b.** Edge of the sample application area. **c.** Surface near the edge of mica. Samples in Figs. a–c contained 1 M urea. **d.** Titin sample with no urea added. **e.** AFM image comparing the global structure of overstretched titin molecules in 0 mM (i) and 1 mM (ii) urea.

**Figure 3:**
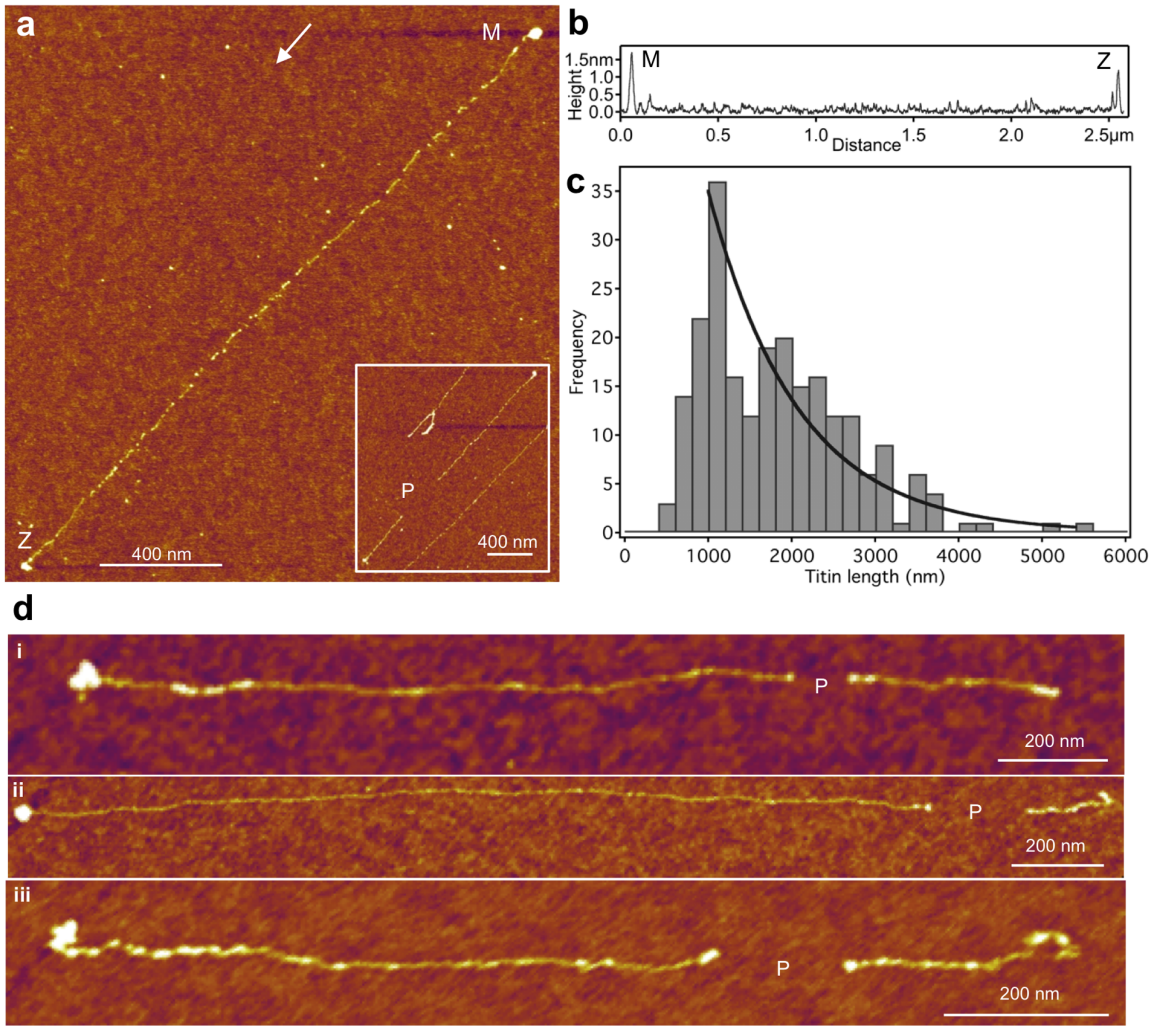
Length analysis of fully straightened and extended titin molecules. **a.** High-resolution AFM image of a titin molecule. *M* and *Z* point at the globular heads in the respective sarcomeric locations, and the arrow indicates the direction of the receding meniscus. **Inset,** example of an overstretched titin molecule containing a large topographical gap that corresponds most plausibly to the PEVK domain (*P*). **b.** Topographical profile plot along the molecule’s axis indicating the globular heads. **c.** Length distribution of titin molecules (n = 255). Thick continuous line is a curve fit with the function 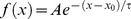, where *A* is frequency maximum, *x_0_* is length offset (1000 nm) and *τ* is decay constant. d. Examples of titin molecules stretched in the presence of 0.6 M KCl. *P* indicates the putative PEVK domain.

**Figure 4:**
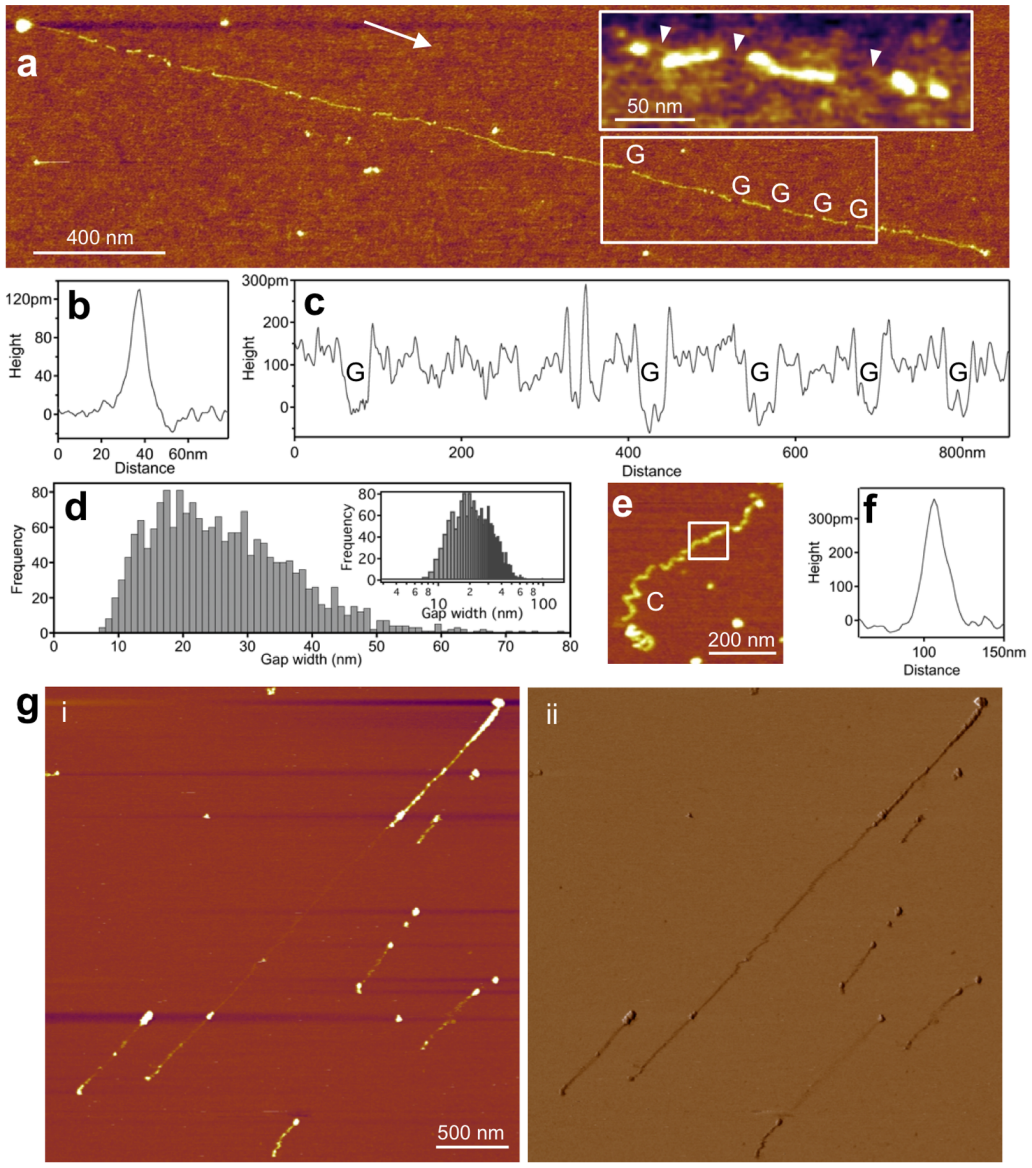
Topography analysis of fully straightened and extended titin. **a.** Example of a stretched titin molecule with topographical gaps (*G*) highlighted. Arrow indicates the direction of the receding meniscus. Boxed area is analyzed in Fig. c. **Inset**, magnified and contrast-enhanced part of an overstretched titin molecule in which fine threads can be discerned in the topographical gaps (arrowheads). **b.** Cross-sectional topography of the extended titin molecule. Average peak height 1.4 Å, width at half maximum height 12.1 nm, and the corrected filament width (equation 1) is 9.3 nm (n = 183). The average cross-sectional profile was measured in a ∼50-nm-long filament region devoid of gaps. **c.** Axial height distribution of the molecule in the region boxed in Fig. a. Topographical gaps are indicated with *G*. **d.** Distribution of gap width (corrected according to equation 2). Mean gap width 27.7 nm (±23.8 nm S.D., n = 1879). Inset, gap-width distribution shown in logarithmic scale. **e.** Example of a conformationally relaxed titin molecule. *C* indicates coiled region of the molecule. **f.** Cross-sectional topography of the relaxed titin molecule in the region boxed in Fig. d. Peak height 3.5 Å, width at half maximum height 13.9 nm, and the corrected filament width (equation 1) is 9.6 nm (n = 51). **g.** Atomic force micrograph of titin recorded under aqueous buffer conditions. Height (i) and phase (ii) contrast images of the same scanned area are shown.

**Figure 5:**
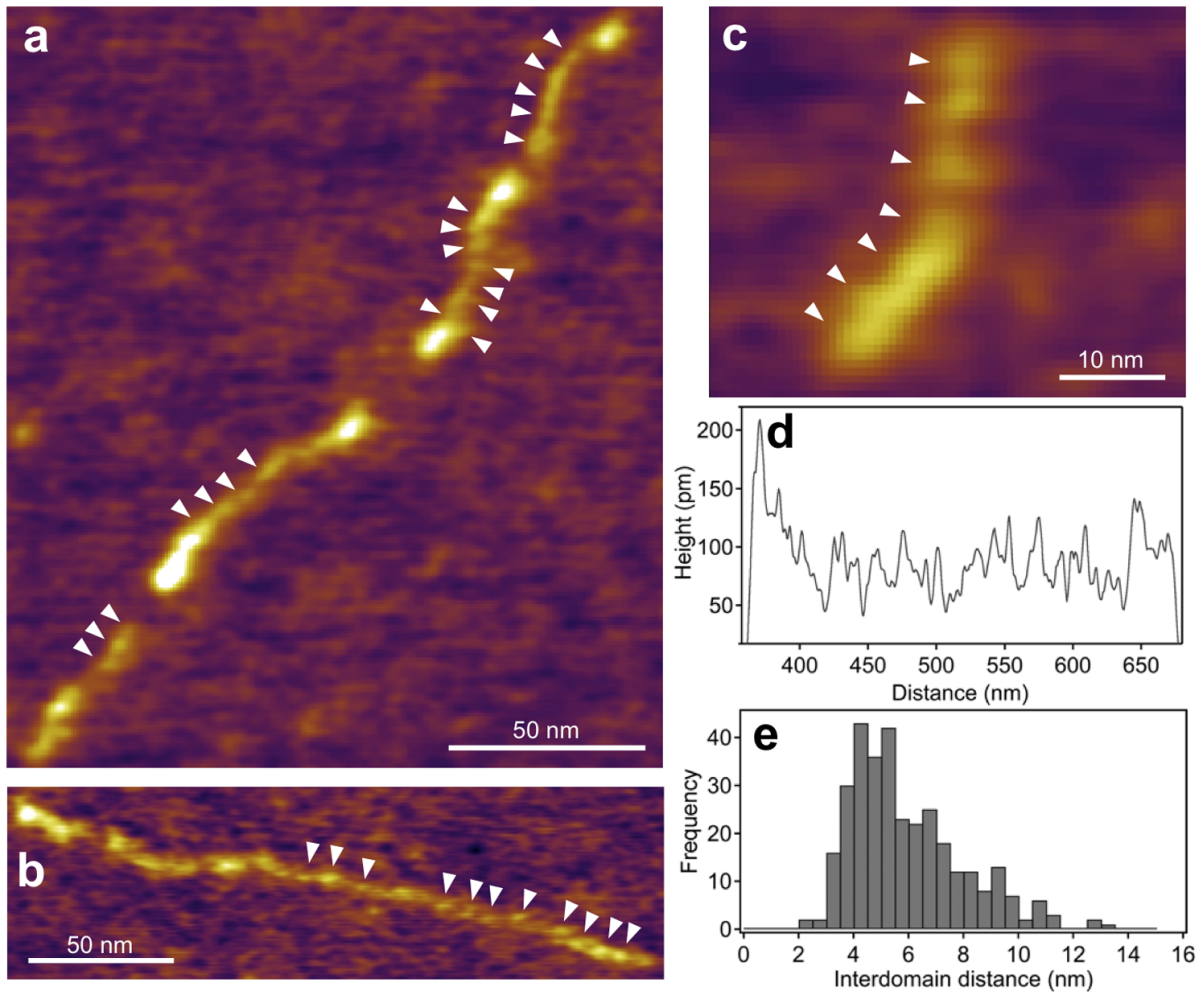
Folded globular domains in titin. **a–c.** Examples of high-magnification AFM images in which ellipsoidal, globular structures can be identified along the contour of the titin molecule. **d.** Example of a topographical height profile along the axis of titin. **e.** Distribution of distance measured between consecutive topographical height peaks. Mean inter-peak distance 5.9 nm (±2.1 S.D., n = 325).

**Figure 6:**
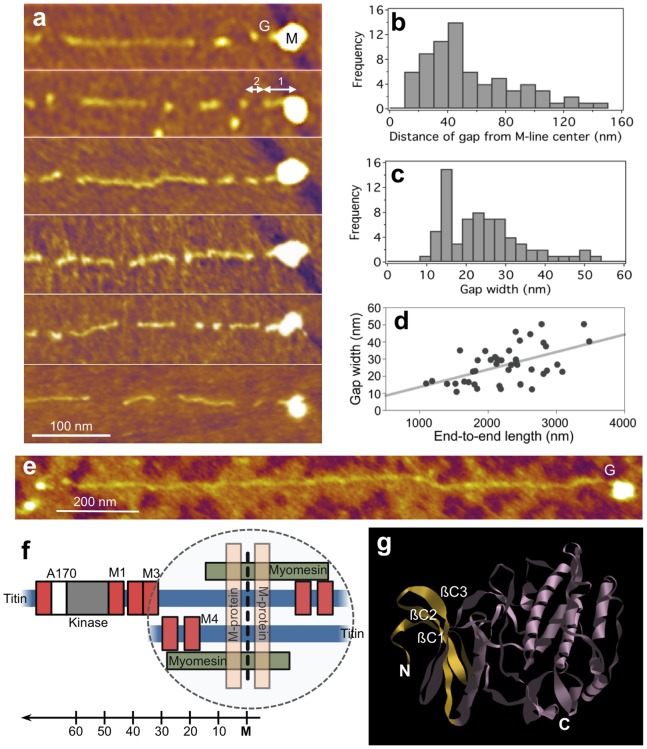
Analysis of domain unfolding near the M-line end of titin. **a.** AFM images of overstretched titin molecules with the M-line end enlarged. *M* and *G* point at the globular M-line head of titin and the topographical gap corresponding to the most distal (i.e., farthest from the N-terminus) unfolded domain, respectively. *1* and *2* indicate the distance of the gap from the M-line center and the gap width, respectively. **b.** Distribution of the distance of the gap from the center of the M-line titin head. **c.** Distribution of gap width. **d.** Width of the distal gap as a function of end-to-end length. Linear fit, correlation coefficient (*r*) is 0.52. e. AFM image of a tiitn molecule stretched in the absence of urea. *G* indicates the most distal gap. **f.** Schematics of the molecular complex in the M-line (cf. [41]). Scale indicates distance, in nanometers, from the center of the M-line. **g.** Molecular model of the titin kinase domain (1TKI). The three N-terminal ß-strands (ßC1, ßC2, ßC3) are highlighted in yellow.

Skeletal-muscle titin molecules, prepared from rabbit *m. longissimus dorsi*, were stretched by molecular combing with receding meniscus (**Fig. 1**) and their topographical structure was analyzed with AFM (**Fig. 2**). In the center of sample application on the mica surface individual molecules and complex oligomers were observed (**Fig. 2a**), which were in relaxed, more-or-less equilibrated conformation. Towards the periphery of sample application extended molecules and oligomers were seen (**Fig. 2b**), and the region near the edge of the mica surface was populated with straightened and overstretched titin molecules (**Fig. 2c**). These overstretched molecules were the subject of subsequent analysis (**Figs. 3**). A typical overstretched titin molecule is shown in **Fig. 3a**. The molecule is essentially straightened out, and globular heads, a large and a small one, can be seen at either of its ends (**Fig. 3b**). We consistently observed the large head on every titin molecule upstream (i.e., towards center of centrifugal rotation), but the small head was sometime absent. We measured the distance between the two heads so as to obtain the end-to-end length distribution of the overstretched titin molecules. The histogram (**Fig. 3c**) shows a large peak at ∼1000 nm and a decaying distribution towards increasing lengths, and the average end-do-end length was 1836 nm (n = 227). Lengths exceeding 5000 nm were also observed. The distribution was well fitted with an exponential function. The topographical structure of extended titin molecules was studied in height contrast images (**Fig. 4**). Both the extended (**Fig. 4a, b**) and the conformationally relaxed (**Fig. 4e, f**) titin molecules appeared flattened based on comparison of their cross-sectional height and width. Gaps were discerned along the axis of the overstretched titin (**Fig. 4a, c**) that interrupted its contour, and which were distributed more or less evenly along the molecule. Occasionally we resolved fine filamentous structures within the gaps (**Fig. 4a** **inset**). Mean gap width was 27.7 nm (±23.8 nm S.D., n = 1879). The gap-width histogram (**Fig. 4d**) displayed a log-normal distribution with the bulk of the data ranging between 6–60 nm and a peak centered around 20 nm. In conformationally relaxed molecules we sometimes observed corkscrew-shaped regions (**Fig. 4e**). In high-magnification AFM images we resolved periodically positioned globular structures along titin’s contour (**Fig. 5**). This topographical periodicity was analyzed by measuring the distance between consecutive peaks in the axial contour plot (**Fig. 5d**). The distribution of the distance between consecutive topographical maxima is shown in **Fig. 5e**. The distances ranged between 2–13 nm, and the average was 5.9 nm (±2.1 nm S.D., n = 325). To obtain further structural insight into the overstretched titin molecule, we analyzed the structural detail in the vicinity of the molecule’s upstream end which is capped with a large globular head (**Fig. 6**). A topographical gap consistently appeared a short distance downstream of the globular head (**Fig. 6a**). The distribution of the distance from the center of the globular head to the edge of the first gap ranged between 10–150 nm with a peak at approximately 50 nm (**Fig. 6b**). The gap-width histogram displayed a sharp peak at 15 nm and a wide one centered at 25 nm (**Fig. 6c**). We found a loose positive correlation (r = 0.52) between gap width and the end-to-end length of the corresponding molecule (**Fig. 6d**).

## Discussion

Individual titin molecules, abundant and extensible protein components of the striated-muscle sarcomere, were overstretched in the present work to lengths up to six times that of the relaxed titin molecule by using molecular combing with receding meniscus (**Fig. 1**) [23]–[25]. Molecular combing has been successfully applied for the investigation of filamentous biomolecules [23], [24], [36], [37], and it provides means to investigate oriented and mechanically extended chainlike molecules and access their conformationally hidden regions.

We observed a gradient of chain conformations from the point of sample application on the mica surface towards the edge of the substrate: whereas conformationally relaxed molecules and complexes dominated the site of sample application, oriented, straightened and overstretched molecules populated the substrate edge (**Fig. 2a–c**). We explain this phenomenon by a competition between the diffusion-driven surface immobilization of titin molecules and the stretching effect of the receding meniscus. Thus, at the site of sample application there is ample time for titin to diffuse to the mica and equilibrate on its surface. By contrast, towards the mica edge titin molecules bind to the surface while the meniscus is already receding and applying a stretching force. The length of the overstretched titin molecules far exceeded that of native titin (∼1 µm, [23], [24]) even by initial visual inspection. In most experiments 1 M urea was added to reduce aggregation. Such a low concentration of urea has been shown before not to cause titin domain unfolding [28]. Indeed, the global appearance of titin molecules overstretched in 0 M (**Fig. 2d**) or 1 M urea (**Fig. 2c**) was similar, and their mean length was not significantly different (**Fig. 2e**, 2103.4±149.3 nm S.E.M. *versus* 2158.7±92.7 nm S.E.M. in 0 and 1 M urea, respectively). However, a more detailed analysis revealed (see **Fig. 2e** and discussion below) that the use of 1 M urea led to the appearence of more numerous but smaller topographical gaps than meniscus force alone. Thus, although 1 M urea reduced aggregation, provided superior image quality and allowed us to gain insight into mechanically-driven local structural changes of titin, its chemical effects must also be taken into account when interpreting our observations.

We systematically observed a large (height ∼10 times that of the rest of the filament) globular head at one end of the molecule thereby confirming earlier observations [23]–[25]. Thus we identify this part of the molecule as its C-terminus that extends into the M-line. The globular M-line-end head contains, in addition to overlapping M3–M5 domain regions of anti-parallel titin molecules from opposite sides of the sarcomere, a dimer of myomesin and M-protein (see schematics in **Fig. 6f**) [38]–[41]. The M-line globular head was consistently observed upstream, towards the center of centrifugal rotation, suggesting that this is the initial point of titin’s contact with the mica surface, and that the M-line-end to mica interaction precedes the stretching action of the receding meniscus. Our results thus support prior observations and a calculation that titin’s M-line end is positively charged at physiological pH values [24], making it the most probable site of binding to the negatively charged surface of mica. Furthermore, the consistent appearance of the M-line-end globular head indicates that it is a stable structure capable of withstanding considerable mechanical forces. Frequently we observed a smaller globular head also at the other end of the molecule which likely corresponds to its N-terminus that extends into the Z-disk (**Fig. 3a–b**). Because we hypothesized that filaments with globular heads at both ends represent the complete titin molecule, further length analysis was carried out on molecules with such feature (**Fig. 3c**).

The length histogram of overstretched titin molecules displayed an overall exponential distribution with an initial maximum at 1000 nm that corresponds well to the length of native titin [23], [24]. The exponential length distribution indicates that the overstretch of titin occurred at the expense of independently unfolding globular (Ig or FN) domains. Furthermore, the titin molecules across the edge of the mica surface and the domains along a titin molecule were exposed to similar forces for comparable time periods. We observed that a marked PEVK-domain extension was rather infrequent. Only about 2% of the analyzed molecules contained large contour interruptions (**Fig. 3a** **inset**) which are thought to correspond to the presence of unfolded and extended PEVK domain [24]. A plausible explanation for this finding is that at the relatively low ionic strength (∼150 mM) employed in our experiments the PEVK domain is probably electrostatically trapped on the mica surface in a contracted conformation. The relative infrequency of the appearance of the PEVK domain in combed titin was also noted in electron microscopy experiments [25]. At elevated ionic strengths and upon replacing NaCl with KCl the frequency of overstretched molecules displaying a wide gap, corresponding most likely to the extended PEVK domain, increased significantly (**Fig. 3d**). Potassium ions have been shown before to compete with the binding of the protonated ε-amino groups of the lysine residue to mica [42]. Thus, inhibition of the rapid binding of PEVK’s numerous lysine residues to mica allowed the extension of this domain prior to its surface capture.

The structural hallmarks of globular-domain unfolding were investigated by topographical analysis of high-resolution (0.5–2 nm pixel resolution) AFM images. AFM provides an advantage over the previously employed analysis methods of molecular combing, because molecular topography is directly accessible without the compromising effect of shadowing, in which the grain size is ∼4 nm [24], [25]. We observed distinct gaps in the titin filaments that interrupted the axial contour (**Fig. 4**). A gap was hereby defined as an axial interruption the bottom of which is in plane with the substrate surface. Similar gaps have been observed in overstrechted titin molecules directionally shadowed and observed by using electron microscopy [25]. The average gap width was 27.7 nm (±0.5 nm S.E.M.), which compares well with the contour length of an unfolded globular domain in titin [11]. Thus, the gaps are the apparent morphological manifestations of individual domain unfolding events, further supporting the notion that domains unfold independently upon the action of mechanical force. Notably, the omission of urea resulted in increased gap width (35.6±3.2 nm S.E.M.) and mean inter-gap distance (134.8±9.2 nm S.E.M. versus 89.5±2.1 nm S.E.M. in 0 and 1 M urea, respectively). Occasionally we observed fine filamentous structures spanning the gaps (**Fig. 4a** **inset**), which most plausibly correspond to the extended protein chain of the mechanically unfolded domain. Notably, we sometimes observed corkscrew-shaped regions in the unstrained titin molecules, suggesting that torsional stress is trapped in the chain prior its final binding to the surface [25]. Much further work is necessary to understand the role of torsional forces in titin nanomechanics. Although here we assign the mean gap width to the extension of individual unfolded globular domains, the large width (bulk of data between 6 and 60 nm) of the gap-width distribution (**Fig. 4d**) and the urea effect suggest that additional molecular mechanisms may also be involved. The short gap widths may be caused by partial or two-step domain unfolding [43], and the large gap widths may correspond to the unfolding of multiples of neighboring domains. Further experimentation will sort out the details of the unfolding mechanisms and the significance of the location, along titin’s axis, of the individual domain unfolding events.

From the ratio of mean topographical height and width we noted that a considerable structural flattening took place in the case of both the overstretched and relaxed titin molecules as their final configurations were estabished on the mica surface (**Figs. 4b and f**). Such a flattening is caused by molecule-surface interactions, dehydration and pressure by the AFM tip [44]. The good correlation between the present titin length measurements and prior electron microscopy data [23]–[25] suggest, however, that although surface interactions caused structural distorsions laterally, axial distorsions remained minimal. To test whether dehydration may have caused the global and local structural changes in the stretched titin molecules, we carried out AFM imaging on hydrated samples, under aqueous buffer conditions (**Fig. 4g**). We could observe overstretched titin molecules on the mica surface. The stretched molecules appeared segmented, which was particularly well visible in phase images that are highly sensitive to local viscoelasticity. Thus, it appears that the overall structure of titin is established during the receding of the meniscus, although additional structural consolidation may take place at the microscopic level during dehydration.

We used three different strategies to estimate the overstretching force acting on titin. Meniscus force may be calculated *a priori* (see **Fig. 1b**) based on the surface tension at the 50% aqueous glycerol - air interface (64 pNnm^−1^ [24]) and the cross-sectional geometry of titin. Assuming that meniscus force acts on a titin molecule in which the globular domains are straightened by viscous drag, a diameter of 2 nm, which corresponds to the average width of an Ig- or FN-type domain, is appropriate [45]. As a result, 400 pN stretching force is calculated. In a second approach, one may *a posteriori* calculate the stretching force from the wormlike-chain equation (equation 4) based on the mean end-to-end distance of unfolded domains (27.7 nm, see **Fig. 4**), the mean contour length of an unfolded globular titin domain obtained from sequence data (32.1 nm, see Materials and Methods) and a persistence length of 0.4 nm for unfolded protein [11]. As a result, 144 pN of stretching force is obtained. Finally, the stretching force may be estimated from the number of domains unfolded during the time window (*Δt*) of receding-meniscus travel along the titin molecule and considering the statistical nature of force-induced unfolding [9], [33]. The average number of domains unfolded during a receding meniscus experiment was obtained from either the gap number statistics (22 unfolded domains/molecule) or the length gain (in excess of 1 µm) normalized by the end-to-end length of an unfolded domain (29 unfolded domains/molecule). Using a time window of 5×10^−6^ s, a spontaneous domain unfolding rate of 3×10^−5^ s^−1^ [33], and an unfolding potential width of 0.3 nm [9], [33] stretching forces of 276 pN and 280 pN are obtained, respectively. The calculated stretching forces (144–400 pN) are thus much smaller than previously estimated [24] and on par with those accessible in dynamic force spectroscopy, making molecular combing a valuable complement to conventional single-molecule mechanics.

Whereas the gaps along the topographical contour of overstretched titin are identified here as regions of domain unfolding, the rest of the contour corresponds to folded molecule. We resolved globular units in these regions the periodicity (mean 5.9 nm) of which corresponds well to the spacing of titin’s globular domains [45], [46]. Given that the axial length of a titin globular domain is approximately 4 nm [30], the 5.9-nm average interdomain distance suggests that there is a nearly 2-nm-long linker between the domains which may confer additional flexibility to the native titin chain.

Considering that the topographical analysis presented here allows to identify and locate folded and unfolded titin regions, we set out to identify the first unfolded region nearest the M-line head (**Fig. 6**). The distribution of distance of the gap from the center of the M-line head (**Fig. 6b**) displays a maximum at ∼50 nm, which corresponds well to the distance of the kinase domain from the M-line center (**Fig. 6f** [40]). Although a sequence-specific identification, with monoclonal antibodies, for example, is currently not available, based on the high-resolution topographical distance mapping we hypothesize that the gap located nearest the M-line head corresponds to the unfolded N-terminal part of the titin kinase. In support, the maxima in the gap-width histogram (**Fig. 6c**) at 15 and 25 nm are comparable to the location of the first two peaks in the single-molecule force spectra of the kinase domain [26]. These force peaks have been associated with the force-driven unfolding of the N-terminal ß-strands (**Fig. 6g**), leading to the opening of the ATP-binding pocket. The presence of this topographical gap in the majority of the overstretched titin molecules indicates that the N-terminal domain of the kinase domain systematically unfolds upon exposure to the receding meniscus. The gap near the M-line end was also well observable in experiments where urea was omitted from the buffer solution (**Fig. 6e**). Considering that on average only 22 globular domains (only 15 domains at 0 M urea) became unfolded per titin molecule upon receding-meniscus action, the systematic unfolding in the kinase makes it one of the mechanically weakest titin domains with canonical structure and supports its role as a discrete mechanosensor. The weak positive correlation between the width of the first gap and the length of the corresponding titin molecule (**Fig. 6d**) suggests that the unfolded N-terminal region of the kinase domain responds to the overall extension of titin, which may have implications on the diffusional access to the ATP-binding pocket. Whether titin kinase might function as a continuous mechanosensor rather than a discrete one only, needs much further investigation. Altogether the topographical distance mapping employed here may allow us to allocate further unfolded domains downstream of the kinase and explore the presence of any spatial pattern in the force-driven structural changes in titin.

## Conclusions

Titin molecules overstretched by receding meniscus and captured on mica surface were visualized here with high-resolution AFM. Periodically arranged globular structures along the molecule’s contour were identified as individual folded domains, and topographical gaps were correlated with unfolded and extended domains. Based on topographical distance mapping we hypothesize that the gap nearest the M-line end corresponds to the N-terminal part of the kinase domain, raising further implications of this domain in sarcomeric mechanosensing.
